# Tri‐trophic interactions among *Fopius arisanus*, Tephritid species and host plants suggest apparent competition

**DOI:** 10.1002/ece3.9742

**Published:** 2023-01-11

**Authors:** Laura Moquet, Benoit Jobart, Romuald Fontaine, Hélène Delatte

**Affiliations:** ^1^ CIRAD, UMR PVBMT Saint‐Pierre Réunion; ^2^ FDGDON Saint‐Paul Réunion; ^3^ CIRAD UMR PVBMT Antananarivo Madagascar

**Keywords:** *Bactrocera dorsalis*, *Bactrocera zonata*, biological control, fruit flies, host range, parasitoid, Tephritidae

## Abstract

When several polyphagous herbivore species share a parasitoid, the tri‐trophic interaction networks can be difficult to predict. In addition to direct effects, the parasitoid may influence the herbivore community by mediating indirect interactions among hosts. The plant species can also modulate the parasitoid preference for a specific host. One of the indirect effects is apparent competition, a negative interaction between individuals as a result of the action of shared natural enemies. Here, we focus on the interactions between the parasitoid *Fopius arisanus* (Braconidae) and two generalist fruit fly pests: *Bactrocera dorsalis* and *Bactrocera zonata* (Tephritidae). This parasitoid was introduced into La Réunion in 2003 to control populations of *B. zonata* and can also interact with *B. dorsalis* since its invasion in 2017. Our main objective is to characterize the tri‐trophic interactions between *F. arisanus*, fruit fly and host plant species. We developed a long‐term field database of fruit collected before and after the parasitoid introduction and after the *B. dorsalis* invasion in order to compare parasitism rate and fruit fly infestation for the different periods. In laboratory assays, we investigated how the combination of fruit fly species and fruit can influence the preference of *F. arisanus*. In the field, before the invasion of *B. dorsalis*, the parasitism rate of *F. arisanus* was low and had a little impact on the fruit fly infestation rate. After the *B. dorsalis* invasion, we observed an increase in parasitism rate from 5% to 17%. A bioassay showed that females of *F. arisanus* could discriminate between eggs of different fruit fly and host plant species. The host plant species preference changed in relation to the fruit fly species inoculated. Field observations and laboratory experiments suggest the possible existence of apparent competition between *B. dorsalis* and *B. zonata* via *F. arisanus*.

## Graphical abstract

Our main objective was to characterize the tri‐trophic interactions between *Fopius arisanus*, fruit fly and host plant species by long‐term field database and laboratory assays. Results suggest a possible existence of apparent competition between *Bactrocera dorsalis* and *Bactrocera zonata* via *F. arisanus*.

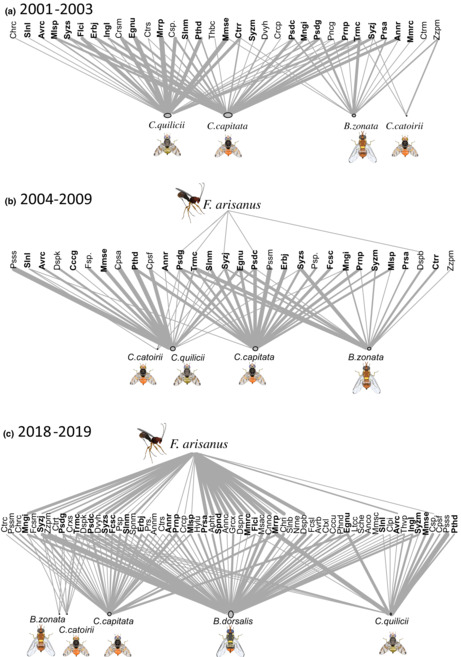

## 1 INTRODUCTION

In the context of human‐induced changes with unintentional (invasion) and voluntary (biological control) alien species introductions, new interactions between species have become frequent and can impact the ecological networks. Studying the ecological mechanisms underlying novel species interactions is a significant challenge to understanding fluctuation in population and community assemblage, such as species colonization and range expansion (Strauss et al., 2006; Wang et al., 2013). However, the ecological outcomes of species interactions can only be fully understood after considering the multi‐trophic approaches in which the species are embedded, i.e. beyond the simple pairwise interactions, the emergent features of interactions visible at least at a tri‐trophic should also be considered (Fortuna et al., 2012; Harvey et al., 2003; Perović et al., 2018; Price et al., 1980; Singh, 2003). Understanding multi‐trophic interactions are fundamental in the context of biological control and pest invasions (Schulz et al., 2019; Tylianakis & Binzer, 2014). For example, the fluctuation of pest herbivore populations can be mediated by resource availability and presences of natural enemies (parasitoids, predators, or pathogens). In return, plants can affect how natural enemies impact herbivore populations (Abdala‐Roberts et al., 2019; Price et al., 1980).

However, the tri‐trophic interaction networks (parasitoid – herbivores – host plants) can be complex and difficult to predict. In addition to the direct negative effect of parasitism, the parasitoid may influence the host species' community structure by mediating negative or positive indirect interactions among hosts (Abrams et al., 1996; Chaneton & Bonsall, 2000; van Veen et al., 2006). Apparent competition refers to an indirect negative interaction between individuals due to the action of shared natural enemies (Bonsall & Hassell, 1997; Holt & Bonsall, 2017; van Veen et al., 2006). Apparent competition can occur when the presence of one prey species increases predator density, thus increasing predation on other species (Density‐dependent indirect effects, Holt & Lawton, 1993; Long et al., 2012). Moreover, apparent competition can occur when the presence of one prey species induces changes in predator traits or behavior, which alter the interaction of the predator with other prey species (trait‐mediated indirect interactions, Werner & Peacor, 2003; Banerji & Morin, 2014). One mechanism underlying these effects is predator or parasitoid selectivity. If the two host species are not equivalent or if the parasitoid has a host preference, the preferred prey species is likely to become extinct (Chailleux et al., 2014; Chaneton & Bonsall, 2000; van Veen et al., 2006). In addition, the plant species can modulate the parasitoid preference for a specific host when herbivore hosts are polyphagous (Traine et al., 2021). Although biological control is founded on the concept of trophic interactions, the impact of indirect effects due to parasitoids is largely unexplored.

One example of complex interactions is found between the parasitoid *Fopuis arisanus* (Sonan, 1932) (Hymenoptera: Braconidae) and the two tephritid species: *Bactrocera dorsalis* (Hendel, 1912) and *B. zonata* (Saunders, 1841) (Diptera: Tepritidae). These three species currently coexist in several parts of the world. *F. arisanus* was introduced in many countries for tephritid biological control (Mohamed et al., 2016), and these two *Bactrocera* species are major invasive pest species both present in Sudan, Pakistan, Mauritius, and La Réunion (Abro, 2020; Mahmoud, Abdellah, et al., 2020; Moquet et al., 2021; Sookar et al., 2021). Furthermore, their distribution overlap could increase if we consider climate change and their potential future distribution area, which has been modeled by several authors (De Villiers et al., 2015; Mahmoud, Mohamed, et al., 2020; Ni et al., 2012). However, the dominant species may vary from region to region. *B. zonata* is the dominant species in Sudan (Mahmoud, Mohamed, et al., 2020), while *Bactrocera dorsalis* is the dominant species in La Réunion and Mauritius (Moquet et al., 2021; Sookar et al., 2021). The outcome of the competition is modulated by factors such as climatic tolerance. Indirect effects linked to parasitoids could also influence the interactions between these two species.

In La Réunion, *F. arisanus* was released between 2003 and 2005. The primary purpose of its introduction was to control *B. zonata* detected on the island for the first time in 2000, but also two *Ceratitis* species with economic impact, *Ceratitis quilicii* De Meyer, Mwatawala and Virgilio, 2016 and *Ceratitis capitata* (Wiedemann, 1824) (White et al., 2000). However, after the invasion of *B. dorsalis* on the island in 2017, the ability of the well‐established *F. arisanus* populations to parasitism again its ancestral host was uncertain. With these multiple unintentional (invasion) and voluntary (biological control) species introductions, La Réunion (France) represents a particular area to study how new interactions can impact ecological networks and tri‐trophic interactions. We explored these questions using a long‐term field database of fruit collected before and after the parasitoid introduction and after the *B. dorsalis* invasion (from 1991 to 2009 and 2018 to 2019). In addition, laboratory experiments were carried out to study the tripartite interactions between host plant, fruit fly species and *F. arisanus* in La Réunion (France). First, we analyzed the change in the infestation and parasitism rate since the introduction of *F. arisanus* in 2003. We supposed that the introduction of *F. arisanus* reduced the infestation rate of *B. zonata* and *Ceratitis* species. After the *B. dorsalis* invasion, we hypothesize that indirect interactions among the two main hosts (*Bactrocera* species) via the parasitoid could exist. Secondly, in laboratory experiments, we analyzed interactions between Tephritidae and *F. arisanus* and how the host plant influenced Tephritidae/parasitoid interactions. It was proven that *F. arisanus* could discriminate and choose between fruit‐fly species eggs for oviposition (Ayelo et al., 2017; Bautista & Harris, 1996; Mohamed et al., 2010; Rousse et al., 2006), and we supposed a preference for *Bactrocera* species in comparison to *Ceratitis* species. However, the preference between *B. zonata* and *B. dorsalis* was more challenging to predict. While *B. dorsalis* is the ancestral parasitoids' host, *Fopius arisanus* interacted with *B. zonata* for 14 years in La Réunion (Moquet et al., 2021). From a tri‐trophic viewpoint, we also supposed that the host plant could modulate fruit fly preferences of the parasitoid. Finally, we discussed how field samplings and experimental results suggest an apparent competition between these species.

## 2 MATERIALS AND METHODS

### 2.1 *Fopius arisanus* and historical data of releases

*Fopius arisanus* is an egg‐larval parasitoid species regularly used for the biological control of Tephritidae. The species is native to the Indo‐Malayan region. It is a solitary koinobiont endoparasitoid that attacks the eggs of fruit fly species and emerged from the puparium (Rousse, 2007). It was used as a biological control for the first time in Hawaii in 1946. Then, it was introduced from Hawaii to many other parts of the world, including Africa and the Indian Ocean, to control tephritid pests (Mohamed et al., 2016; Purcell, 1998; Rousse et al., 2005). *Fopius arisanus* can attack numerous fruit fly species, but it predominantly attacks *Bactrocera* species (Mohamed et al., 2010; Rousse et al., 2006; Zenil et al., 2004). In the introduction regions, this generalist species was regularly exposed to several hosts that coexist, for example, *F. arisanus* control *B. dorsalis*, *Bactrocera kirki* (Froggatt, 1911), and *Bactrocera tryoni* (Froggatt, 1897) in French Polynesia (Vargas et al., 2007, 2012). In La Réunion, *F. arisanus* can attack *Bactrocera dorsalis*, *Bactrocera zonata*, and *Ceratitis* species (Rousse et al., 2006).

In La Réunion, the initial colony of *F. arisanus* was established in 2003 in the CIRAD‐3P Réunion Entomology Laboratory from parasitized pupae of *B. dorsalis* obtained from USDA‐ARS Hawaii (E. J. Harris). In the laboratory, the parasitoid was reared on *B. zonata* and then released between December 2003 and May 2005 (Rousse et al., 2006). Approximately 74,800 individuals were released in different parts of the island (Table 1; Quilici et al., 2005).

**TABLE 1:** Sites and dates of releases of *Fopius arisanus* in La Réunion

Zones	Site names	Date	Number	Lat.	Long.
North	Saint Denis, Rivière Saint Denis	07/12/2003	9000	−20.88726	55.45074
North	Saint Denis, Rivière Saint Denis	16/12/2003	2000	−20.88726	55.45074
South	Saint Pierre, Hôpital Terre Sainte	05/02/2004	4500	−21.34670	55.49394
South	Ravine des Cabris, Vieux Domaine	05/03/2004	5500	−21.28493	55.47944
South	Ravine des Cabris, Vieux Domaine	16/03/2004	3200	−21.28493	55.47944
West	L'hermitage, Jardin d'Eden	05/04/2004	3600	−21.07633	55.22936
East	Saint Benoit, Parking du marché	26/04/2004	5000	−21.03371	55.71445
South	Saint Pierre, Hôpital Terre Sainte	12/05/2004	5000	−21.34670	55.49394
South	Ravine des Cabris, Vieux Domaine	26/05/2004	5000	−21.28493	55.47944
South	Ravine des Cabris, Vieux Domaine	23/02/2005	2000	−21.28493	55.47944
South	Ravine des Cabris, Vieux Domaine	30/03/2005	20,000	−21.28493	55.47944
West	Etang Salé	09/05/2005	10,000	NA	NA
Total			74,800		

### 2.2 Field collection

To study interactions among fruit fly and parasitoid species, we performed field campaigns on the entire island of La Réunion. La Réunion is located in the southern Indian Ocean (55°30′E; 21°10′S), around 700 km off the coast of Madagascar. It is a volcanic island that rises to an altitude of 3100 m. Its topography is rugged and has a humid tropical climate, with a dry season from May to October and a wet season from November to April.

Sampling was regularly performed between 2000 and 2003, just after the *B. zonata* invasion, between 2004 and 2009 (except 2008), during and after the release of *F. arisanus* (Duyck et al., 2008) and between 2018 and 2019 after the *B. dorsalis* invasion (Moquet et al., 2021). The same data collection method was used throughout the different sampling periods. We collected ripe fruit samples on the ground or on trees from different plant species (cultivated, ornamental or wild) all over the island. Whenever possible, we sampled 15 fruits for each plant species found per location and date. In total, we collected more than 33,500 individual pieces of fruit from 112 potential host plant species.

In the laboratory, the fruit samples were individually weighed, placed in plastic boxes with sand as pupation substrate, and covered with a fine‐mesh cloth. We put fruit samples in a maturation room (25°C ± 2°C and 70 ± 20% humidity) until pupation. Fruit samples were regularly inspected for 3 weeks, and the sand was sifted to look for pupae. Pupae were kept in a climatic room in plastic boxes until their emergence, when they were taxonomically identified to species level. We identified fruit flies and parasitoids (Appendix S1) using morphological criteria (Virgilio et al., 2014; Wharton & Yoder, 2021). Identification was performed at emergence. Fruit could be infested by several fruit flies and it was impossible to determine which fruit fly species was parasitized.

We recorded the number of emerging individuals for each fruit fly species or parasitoid according to fruit (species and weight), site and date (of collection). We calculated (i) the fruit fly infestation rate as the number of emerged flies per kg of collected fruit and (ii) the parasitism rate as the number of parasitoids on the number of emerged imago (flies and parasitoids). Following other studies on parasitism of fruit flies (Aluja et al., 1990; Dieng et al., 2020; Eitam & Vargas, 2007; García‐Medel et al., 2007; Ovruski et al., 2004), we calculated the parasitism rate (PR) of *Fopius arisanus* for each host plant species separately with the formula: PR_*i*_ = *P*_*i*_/(*P*_*i*_ + FF_*i*_) with *i* a particular host plant species, *P* the number of emerged parasitoids, and FF the number of emerged fruit flies. The global parasitism (PR_G_) rate is defined as the total parasitism rate for all host plant species infested by generalist fruit fly species (*B. dorsalis*, *B. zonata*, *C. capitata*, *C. catoirii*, *C. quilicii*): PR_G_ = ∑ *P*_*i*_/(∑ *P*_*i*_ + ∑ FF_*i*_). Even if *Dacus ciliatus* Loew, 1862, *D. demmerezi* (Bezzi, 1917) and *Neoceratitis cyanescens* (Bezzi, 1923) can be hosts for *F. arisanus* in a laboratory, in La Réunion we did not observe *F. arisanus* in co‐emergence with these species or in their host plants (Curcurbitaceae and Solanaceae), that is why, they were not included in the PR_G_. In addition, to compare the variation of *F. arisanus* abundance over time, we calculated the number of parasitoids per kg of fruit.

In addition, the adult population levels of *Bactrocera* sp. (number of flies/trap/day) were investigated by the analyses of a trap network for epidemiological surveillance (SBT/SORE: Biological monitoring of the territory – Surveillance of regulated or emerging organisms) piloted by the Direction of Food, Agriculture and Forest (DAAF) of La Réunion and carried out by FDGDON. Traps were installed around the island between 2015 and 2016 (before *B. dorsalis* detection), in 2017 (just after *B. dorsalis* detection) and 2022. These traps were “Maxi Trap” type or recycled bottles with Methyl Eugenol to attract males of *Bactrocera* sp. and with an insecticide (Deltamethrine). Their number varied according to the period: 20 traps between 2015 and 2016, 201 traps just after *B. dorsalis* detection, and 10 in 2020 (Appendix S2).

### 2.3 Experimental test

#### 2.3.1 Insects

We used *F. arisanus* from lab‐reared strains to test parasitoid preference for fruit fly species and host plant species. *Fopius arisanus* was reared in the Entomology Laboratory from wild individuals collected in the field on *Terminalia catappa* fruit. One colony of parasitoids was reared on *B. zonata* eggs since 2017, and the other on *B. dorsalis* eggs since 2019. Wild individuals were regularly added to the two colonies.

We tested *F. arisanus* parasitism rate on three tephritid species regularly parasitized by this species in La Réunion: *B. dorsalis*, *B. zonata*, *C. quilicii*. Fly strains were collected from samples of different host plant species from La Réunion and larvae were subsequently fed on an artificial diet (Duyck & Quilici, 2002). Fruit fly eggs used for bioassays were collected from routine rearing cages (housing a few thousand females), into which we placed a perforated plastic ball containing a small piece of fruit (guava, lime, mango, or papaya) to stimulate egg laying inside this oviposition device. Eggs were never rinsed and were manipulated with a fine wet paintbrush.

Parasitoids and flies were reared in a 45 × 45 × 45 cm plastic screened cage at 25 ± 2°C, 70 ± 20% RH, with a 12 L:12D photoperiod. The adults were given free access to water and food consisting of sugar and enzymatic protein hydrolysate.

#### 2.3.2 Fruits

We chose host plant species according to the infestation rates observed in the field in La Réunion for the target tephritid species (Moquet et al., 2021). We selected: (i) two host plants regularly visited by the three fruit flies studied: guava (*Psidium guajava* L.), mango (*Mangifera indica* L.); (ii) one host plant was only visited by *B. dorsalis* in La Réunion: papaya (*Carica papaya* L.); and (iii) one host plant was never visited by fruit flies: lime fruit (*Citrus aurantifolia* L., Moquet & Delatte, 2021). We used ripe fruit with no pesticide treatment. We protected guava and mango with fine‐mesh nylon bags at the unripe stage to avoid infestation by wild fruit flies. We collected unripe papaya and kept it in the laboratory at room temperature until the ripe stage. We visually checked the absence of stings on the limes. To provide a standardized oviposition substrate, fruit samples were cut into small pieces of about 9 cm^2^ with two slits of 5 mm deep to slip in the eggs of fruit flies.

#### 2.3.3 General protocol

We tested whether the oviposition choice of *F. arisanus* was influenced by the host plant and fruit fly species. Using a fine wet paintbrush, we gently deposited 50 <4 h old fruit fly eggs in each slot (100 eggs per fruit). Fruit samples were spaced approximately 10 cm apart and exposed to naïve and mated parasitoid females (4–15 days old) for 24 h in 30 × 30 × 30 cm cages with natural light. At the end of the experiment, we rinsed fruit samples with water and sieved eggs on a piece of thin netting. We dechorionated the eggs using the same protocol as Rousse et al. (2006). Eggs were immersed for 60 s in a 2.6% NaClO solution and then rinsed with water. They were deposited onto a microscope slide with mineral oil and observed under a binocular microscope at 100× magnification. The proportion of parasitized eggs was calculated as the number of parasitized eggs over the total number of counted eggs.

#### 2.3.4 Fruit fly species

To test parasitoid choice according to fruit fly species, we exposed eight *F. arisanus* females to eggs of different combinations of two fruit fly species (*B. dorsalis/B. zonata*; *B. dorsalis/C. quilicii* or *B. zonata/C. quilicii*). We arranged two pieces of guava, one with 100 eggs of one species and the other with 100 eggs of the second species. Each cage constituted a replicate (*n* = 8 for each species combination). We had four experimental blocks in which each combination was tested simultaneously (3 species combination × 2 *F. arisanus* colonies). We also conducted no‐choice tests following the same protocol but using the same species on both pieces of guava (*n* = 5).

#### 2.3.5 Host plant species

To test parasitoid choice regarding host plant species, we exposed 16 *F. arisanus* females to eggs (100 eggs per fruit) deposited on a piece of guava, lime, mango, and papaya, simultaneously. This experiment was carried out with eggs from the three fruit fly species. Each cage constituted a replicate (*N* = 9 for *B. dorsalis*, *N* = 17 for *B. zonata*, *N* = 20 for *C. quilicii*).

### 2.4 Statistical analyses

All analyses were conducted in R (R Development Core Team, 2021), and data are presented as mean ± standard error. When we used Generalized Linear Mixed Models (GLMM), we always checked the homoscedasticity, normality, and independence of residuals graphically.

#### 2.4.1 Field collections

We compared the infestation rate of *B. zonata* and *C. quilicii* (not enough data for doing any statistical analysis for *C. capitata* using infestation rates) before *F. arisanus* releases (from 2001 to 2003), after the parasitoid release (from 2004 to 2009), and after the detection of *B. dorsalis* (from 2018 to 2020). Furthermore, we studied the variation of the parasitism rate of *F. arisanus* just after its introduction and after the invasion of *B. dorsalis*. We used GLMM adapted for zero‐inflated data with negative binomial to test, for each host plant, the effect of the studied period on the infestation rate and parasitism rate (function “glmmTMB”, package ‘glmmTMB’, Brooks et al., 2017). Fruit batches and host plants were added as random factors. Only observations from fruit samples from the plant species *Psidium cattleianum*, *P. guajava*, *Syzygium jambos*, and *Terminalia catappa* were included in this analysis. These host plants were frequently infested by *B. zonata* and *B. dorsalis*, had broad distribution on the island, and were regularly collected during the three studied periods. In addition, indices from a matrix representing the interactions observed between fruit fly species (columns) and host plant species (rows) for these three periods were calculated. We choose only species present in all three periods to facilitate comparison (30 species). The function “networklevel” and “specieslevel” of the ‘bipartite’ package (Dormann et al., 2008, 2009) were used to determine indices describing networks (connectance, links per species, cluster coefficient, nestedness, H2’, C.score) and species properties in the network (degree, normalized degree, species strength, weighted closeness). We designed the food web analysis for each period with the package ‘igraph’ (Csardi & Ant, 2006) from a matrix of interactions among host plants and emerging insects. Nodes were arranged in the form of a tree according to the Sugiyama layout algorithm, where *F. arisanus* species was used as the root.

#### 2.4.2 Experimental test

Generalized Linear Mixed Models was used to test the effect of fruit fly species on the proportion of parasitized eggs during the choice experiment. The influence of fruit fly species in each species combination (species: combination, with combinations *B. dorsalis/C. quilicii*, *B. zonata/C. quilicii*, *B. dorsalis/B. zonata*) and the colony of *F. arisanus* were fixed factors, and the cage was a random factor. We used a simplified model (GLM) with fruit fly species and the colony of *F. arisanus* (fixed factors) for the no‐choice experiment. When one factor had a significant effect (*p* < .05), pairwise comparisons of values of least‐square means across groups (“lsmeans” command) were computed as a post hoc test with the Tukey HSD method for adjusting *p* values.

Similarly, we performed a GLMM to test the influence of host plant species on the proportion of eggs parasitized by *F. arisanus*. In this case, the proportion of parasitized eggs was the response variable; we tested the influence of host plant species, fruit fly species, and the colony of *F. arisanus* (fixed factors). The interactions between fruit fly species and host plant species were also tested. We added cages as a random factor.

## 3 RESULTS

### 3.1 Field collection

From 2005, *F. arisanus* was regularly found in samples across the island. Between 2005 and 2009, before the invasion of *B. dorsalis*, the mean infestation rate varied from 0.7 ± 0.2% for *P. cattleianum* to 11.5 ± 0.5% for *T. catappa*. We observed parasitoid emergence in only five host plant species among the 25 plant species infested by *B. zonata*, *C. quilicii*, or *C. capitata* (*Diospyros blancoi*, *P. cattleianum*, *P. guajava*, *S. jambos*, and *T. catappa*). The global parasitism rate was only 0.3% in 2005 and fluctuated between 4.7% in 2007 and 8.6% in 2006 and 2009, respectively. We did not observe a significant difference in infestation rates of *B. zonata*, and *C. quilicii* before or after the introduction of *F. arisanus* (df = 8242, *t* = −0.529; *p* = .857 for *B. zonata* and df = 8252, *t* = −1.477, *p* = .302 for *C. quilicii*). Network and species indicess were similar between these two periods (Table 2).

**TABLE 2:** Indices calculated on bipartite networks between fruit flies and host plant species in La Réunion between 2001 and 2003 before the introduction of *F. arisanus*, between 2004 and 2009 after the introduction of *F. arisanus* and, in 2018–2019 after the introduction of *B. dorsalis*

	Indices	2001–2003	2004–2009	2018–2019
Network indexes	Connectance	0.55	0.53	0.48
	Links per species	1.89	1.81	2.00
	Cluster coefficient	0.65	0.61	0.52
	Nestedness	22.79	16.53	13.08
	H2’	0.34	0.32	0.35
	Fruit flies: C.score	0.36	0.24	0.20
	Host plants: number of species	24	22	25
*C. catoirii*	Degree	3	4	3
	Normalized degree	0.13	0.18	0.12
	Species strength	0.43	0.34	0.00
	Weighted closeness	0.01	0.00	0.00
*C. quilicii*	Degree	19	16	16
	Normalized degree	0.79	0.73	0.64
	Species strength	11.21	10.28	5.28
	Weighted closeness	0.46	0.53	0.23
*C. capitata*	Degree	19	14	13
	Normalized degree	0.79	0.64	0.52
	Species strength	8.65	4.68	5.06
	Weighted closeness	0.20	0.03	0.02
*B. zonata*	Degree	12	13	6
	Normalized degree	0.50	0.59	0.24
	Species strength	3.72	6.70	0.06
	Weighted closeness	0.61	0.76	0.02
*B. dorsalis*	Degree	–	–	22
	Normalized degree	–	–	0.88
	Species strength	–	–	14.60
	Weighted closeness	–	–	0.99
*F. arisanus*	Degree	–	5	19
	Normalized degree	–	0.22	0.73

*Note*: Only the common 30 plant species collected during the three periods were used for analyses. See Dormann et al. (2008, 2009) for description of indices.

**FIGURE 1:**
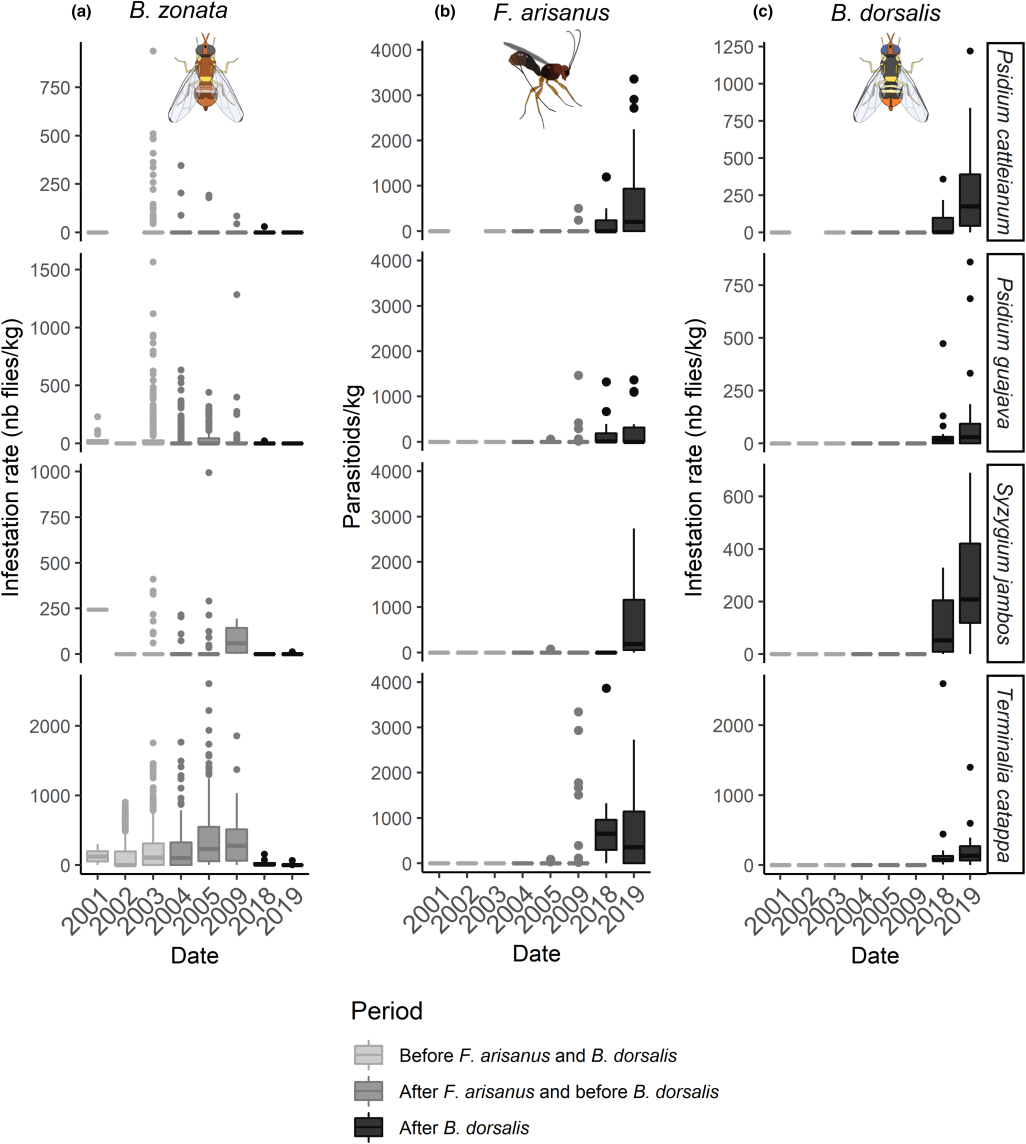
Variation in time of (a) infestation rate of *B. zonata* (b) number of *F. arisanus* per kg and (c) infestation rate of *B. dorsalis* in La Réunion in relation to four main host plant species (*Psidium cattleianum*, *Psidium guajava*, *Syzygium jambos*, and *Terminalia catappa*). Three periods were chosen: 2001–2003 and 2004–2009, which correspond to before and after the introduction of *F. arisanus*, respectively, and 2018–2019, after the introduction of *B. dorsalis*.

In 2018–2019, after the *B. dorsalis* invasion, the parasitism rate of *F. arisanus* significantly increased (df = 5061, *Z* = −2.151, *p* = .031, Figure 1) and reached 16.4 ± 1.2% for *S. jambos*, 18.75 ± 0.22% for *P. cattleianum*, 23.5 ± 1.0% for *P. guajava* and 37.2 ± 1.6% for *T. catappa* (Table 3). The global parasitism rate (PR_G_) was 17.0% for this period, and the number of links (degree) in comparable networks increased from 5 to 19 (Table 2). Moreover, we observed a significant decrease in the infestation rate of the three fruit fly species after the *B. dorsalis* invasion (Figure 1; df = 8242, *t* = −4.704; *p* < .001 for *B. zonata*; df = 8252, *t* = −5.966; *p* < .001 for *C. quilicii*). Moreover, after the *B. dorsalis* invasion, the network indices were impacted: the cluster coefficient, the nestedness, and the C‐score decreased. Species strength decreased for *C. catoirii*, *C. quilicii*, and *B. zonata* (Table 2).

**TABLE 3:** Parasitism rate of *Fopius arisanus* on different infested host plants in La Réunion between 2018 and 2019.

Family	Latin name	*N*	Weight (g)	Pupae number	Number of emerged flies	Fruit fly species	Parasitism rate (%)	Abv
Anacardiaceae	*Anacardium occidentale*	15	1165.5	133	86	B. dor	0	Anco
	*Mangifera indica*	244	61409.9	4266	2381	B. dor; B. zon; C. qui	17 ± 3	Mngi
	*Spondias dulcis*	42	2430.7	42	17	B. dor	40 ± 24	Spnd
	*Spondias mombin*	60	738.3	44	32	B. dor; C. qui	16 ± 11	Spnm
Annonaceae	*Annona muricata*	9	4042	19	12	B. dor; C. qui	0	Annm
	*Annona reticulata*	19	3622	66	61	B. dor; C. qui	0	Annr
	*Cananga odorata*	75	241.3	23	13	B. dor	41 ± 17	Cnno
Aphloiaceae	*Aphloia theiformis*	75	144.8	9	4	B. dor	30 ± 20	Apht
Apocynaceae	*Cascabela thevetia*	121	1936.2	89	44	B. dor; C. cap	0	Thvp
Arecaceae	*Phoenix dactylifera*	45	592	1	1	B. dor	0	Phnd
Bromeliaceae	*Ananas comosus*	13	6808	18	10	B. dor	19 ± 10	Annc
Cactaceae	*Hylocereus undatus*	13	4706	296	203	B. dor	0	Hylu
Caricaceae	*Carica papaya*	35	19,931	152	74	B. dor; C. qui	24 ± 11	Crcp
Chrysobalanaceae	*Chrysobalanus icaco*	15	216.2	79	27	B. dor	40 ± 13	Chri
Clusiaceae	*Calophyllum inophyllum*	30	996.8	5	3	B. dor; C. cap	0	Clpi
	*Garcinia xanthochymus*	8	900	63	32	B. dor	8 ± 8	Grcx
Combretaceae	*Terminalia catappa*	588	19381.5	5657	2726	B. dor; B. zon; C. cap; C. cat; C. qui	37 ± 2	Trmc
Cucurbitaceae	*Coccinia grandis*	120	1274.9	405	260	Z. cuc; D. cil	0	‐
	*Cucumis sativus*	15	2192	189	176	Z. cuc; D. cil; D. dem	0	‐
	*Cucurbita moschata*	56	893.7	537	307	Z. cuc; D. cil; D. dem	0	‐
	*Cucurbita pepo*	30	2561	69	60	Z. cuc; D. cil	0	‐
	*Lagenaria siceraria*	16	4486.4	66	44	Z. cuc; D.dem	0	‐
	*Momordica charantia*	311	3559.9	1109	601	B. dor; Z. cuc; D. cil; D.dem	0	Mmrc
	*Sechium edule*	118	22841.8	203	127	B. dor; D. cil; D. dem	0	Sche
Ebenaceae	*Diospyros blancoi*	15	3422.7	846	478	B. dor	16 ± 7	Dspb
	*Diospyros kaki*	135	10456.6	273	132	B. dor; B. zon; C. cap; C. qui	2 ± 01	Dspk
	*Diospyros nigra*	75	5602.5	13	6	B. dor	40 ± 40	Dspn
Fabaceae	*Inga laurina*	30	691.2	20	16	B. dor; C. cap	0	Ingl
	*Pithecellobium dulce*	30	275.3	3	3	C. cap	0	Pthd
Lauraceae	*Persea americana*	73	23815.1	216	164	B. dor	5 ± 4	Prsa
Moraceae	*Ficus carica*	60	1910.3	305	114	B. dor; C. qui	20 ± 7	Fcsc
	*Ficus lateriflora*	15	100.4	2	2	B. dor	0	Fcsl
	*Ficus mauritiana*	30	1856.4	31	22	C. qui	0	Fcsm
Musaceae	*Musa acuminata*	67	6750.8	632	421	B. dor	0	Msac
Myrtaceae	*Eugenia uniflora*	135	546.3	105	51	B. dor; C. cap; C. qui	13 ± 13	Egnu
	*Psidium catlleianum*	1456	15094.8	2675	1421	B. dor; B. zon; C. cap; C. qui	19 ± 1	Psdc
	*Psidium guajava*	565	28708.5	3469	1804	B. dor; B. zon; C. cap; C. cat; C. qui	24 ± 2	Psdg
	*Syzygium jambos*	615	11027.7	3364	1804	B. dor; B. zon; C. cap; C. cat; C. qui	16 ± 2	Syzj
	*Syzygium malaccense*	25	917.2	33	29	B. dor; C. cap	0	Syzm
	*Syzygium samarangense*	180	3322.3	384	216	B. dor; C. qui	28 ± 4	Syzs
Oleaceae	*Noronhia emarginata*	30	631.9	3	1	B. dor	0	Nrne
Oxalidaceae	*Averrhoa bilimbi*	55	1632.3	1	1	B. dor	0	Avrb
	*Averrhoa carambola*	63	4401.6	8	5	B. dor; C. cap	38 ± 24	Avrc
Passifloraceae	*Passiflora tripartita*	60	2614.2	164	47	C. qui	0	Pssm
	*Passiflora suberosa*	186	141.95	141	106	C. cap	0	Psss
Polygonaceae	*Coccoloba uvifera*	135	268.4	3	3	B. dor	0	Cccu
Rhamnaceae	*Ziziphus mauritiana*	105	2428.3	237	169	B. dor; B. zon; C. qui	2 ± 2	Zzpm
Rosaceae	*Eriobotrya japonica*	447	4480.6	688	229	B. dor; C. qui	13 ± 3	Erbj
	*Malus pumila*	23	1015.6	47	23	B. dor; C. qui	6 ± 4	Mlsd
	*Prunus persica*	268	10,008	2216	1056	B. dor; C. qui	11 ± 2	Prnp
	*Prunus sp*.	83	2772.3	300	111	B. dor; C. qui	0	Prns
	*Pyrus sp*.	78	7669	324	113	B. dor; C. qui	0	Pyrs
Rubiaceae	*Coffea sp*.	388	943.2	62	50	C. cap	4 ± 4	Coff
Rutaceae	*Citrus aurantifolia. x Fortunella sp*.	11	354.4	22	17	B. dor	0	Ctrl
	*Citrus clementina*	80	5613.3	13	1	C. qui	0	Ctrc
	*Citrus reticulata x Citrus sinensis*	51	5731.8	39	18	C. qui	0	Ctrs
	*Citrus sinensis*	75	9230	21	16	B. dor; C. qui	0	Crss
	*Citrus tangerina*	104	6281.3	24	4	C. qui	25 ± 25	Ctrt
	*Murraya paniculata*	315	149.45	170	122	C. cap; C. qui	1 ± 1	Mrrp
Salicaceae	*Dovyalis hebecarpa*	75	396.6	15	5	B. dor; C. qui	25 ± 25	Dvyh
	*Flacourtia indica*	123	919.7	29	11	B. dor; C. cap; C. qui	8 ± 8	Flci
Sapindaceae	*Litchi chinensis*	56	1070.7	7	6	B. dor	0	Ltcc
Sapotaceae	*Chrysophyllum cainito*	15	1222	34	17	C. qui	0	Chrc
	*Mimusops coriacea*	75	2497.3	23	14	B. dor	0	Mmsc
	*Mimusops elengi*	59	286.9	4	4	C. cap	0	Mnse
Solanaceae	*Capsicum frutescens*	73	317.5	15	11	C. cap	0	Cpsf
	*Solanum betaceum*	71	2705.1	25	19	B. dor; N.cya	0	Slnb
	*Solanum lycopersicum*	114	2074.8	50	40	B. dor; C. cap; N. cya;	0	Slnl
	*Solanum mauritianum*	645	940.15	73	48	B. dor; C. qui; N. cya;	8 ± 7	Slnm
	*Solanum melongena*	27	3289.3	20	7	N. cya	0	–
	*Solanum nigrum*	117	26.4	18	16	N. cya	0	–
	*Solanum torvum*	60	93.9	8	7	N. cya	0	–

*Note*: See Moquet et al. (2021) and data available in CIRAD dataverse (https://doi.org/10.18167/DVN1/RMQQFZ) for details of infestation rate by fruit flies. *N*: Number of pieces of fruit collected and total weight (g). Fruit fly species emerging in host plants: B. dor: *Bactrocera dorsalis*, B. zon: *Bactrocera zonata*, C. cap: *Ceratitis capitata*, C. qui: *Ceratitis quilicii*, C. cat: *Ceratitis catoirii*, Z. cuc: *Zeugodacus cucurbitae*, D. cil: *Dacus ciliatus*, D. dem: *Dacus demmerezi*, N. cya: *Neoceratitis cyanescens*. Abv: Abbreviation used in Figure 2.

**FIGURE 2:**
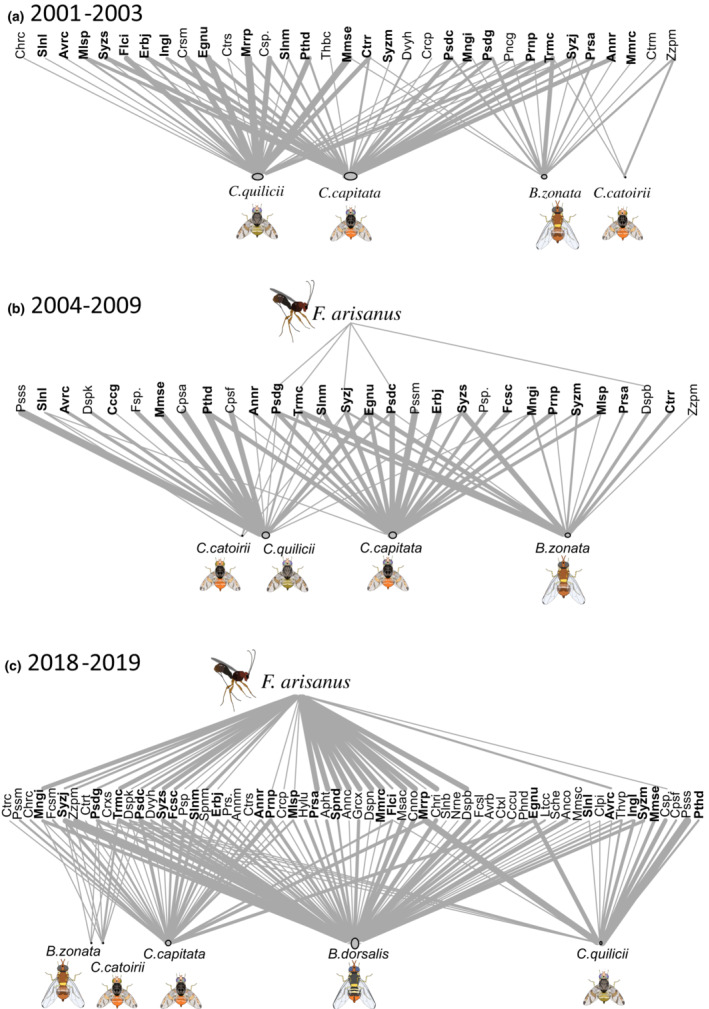
Plot webs representing host plant species and their interactions with the parasitoid *Fopius arisanus* and five fruit fly species in La Réunion (a) between 2001 and 2003 before the introduction of *F. arisanus*, (b) between 2004 and 2009 after the introduction of *F. arisanus* and, in (c) 2018–2019 after the introduction of *B. dorsalis*. Nodes are arranged according to the Sugiyama layout algorithm. Edge width between *F. arisanus* and host plant species are dependent on parasitism rate, edge width between host plant species and fruit flies are dependent on infestation rate and node size is proportional to the degree of the vertices (number of adjacent edges). See Table 3 for abbreviations of host plant species. In bold, host plant species sampled during the three periods.

After the detection of *B. dorsalis* in La Réunion, *F. arisanus* was the most abundant parasitoid of fruit flies (3012 individuals collected). It emerged from 715 individual fruit from 36 plant species (Table 3, Figure 2). This parasitoid's host plant species were infested by *B. dorsalis*, *B. zonata*, *C. capitata*, *C. catoirii*, or *C. quilicii* (Table 3). Of the 36 host plant species of *F. arisanus*, 30 were host plants for *B. dorsalis*, 20 for *C. quilicii*, 10 for *C. capitata*, 7 for *B. zonata*, and 3 for *C. catoirii* (Figure 2). However, we did not find *F. arisanus* in 32 other host plants infested by these five generalist fruit flies (Figure 2).

In the methyl eugenol traps for epidemiological surveillance, the first months after *B. dorsalis* detection, the number of *B. dorsalis* /trap/day was 0.04 ± 0.00. In 2022, we caught approximately 21.26 ± 18.61 *B. dorsalis* per trap per day. Before *B. dorsalis* detection, the mean number of *B. zonata* per trap per day was 19.87 ± 0.49. Just after *B. dorsalis* detection, the number of *B. zonata* was significantly lower (*p* < .001, see Appendix S2) and was, in mean, 2.68 ± 0.23. In 2022, no *B. zonata* was caught.

### 3.2 Experimental test

#### 3.2.1 Fruit fly species

We did not observe a significant difference in the proportion of parasitized eggs between the colony of *F. arisanus* reared on *B. dorsalis* eggs, and the colony reared on *B. zonata* eggs during choice experiments ($\chi_{1}^{2}$ = .041, *p* = .839).

**FIGURE 3:**
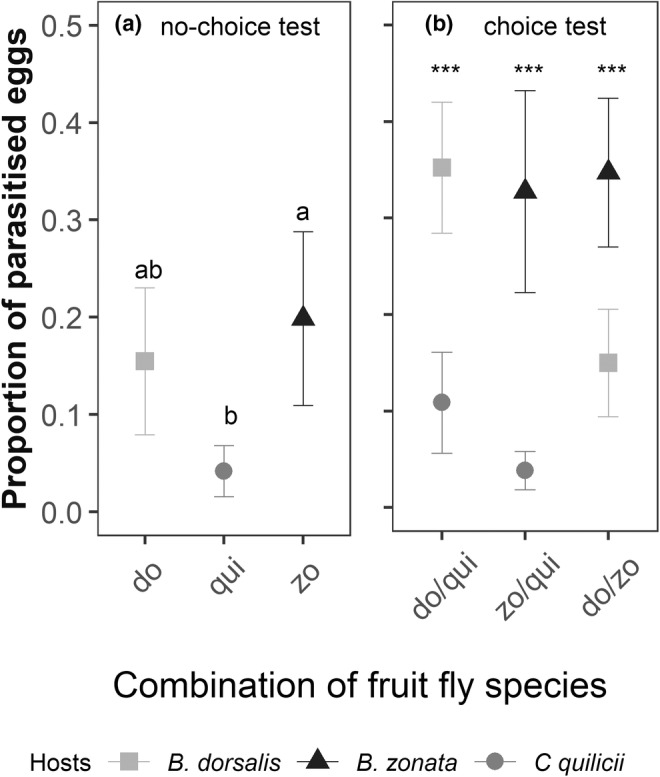
The proportion of parasitized eggs (mean ± SE) by *Fopius arisanus* for eggs deposited in two pieces of *Psidium guajava* for the different fruit fly species and the choice proposed in (a) no‐choice experiment and (b) choice experiment (do: *B. dorsalis*, zo: *B. zonata*, qui: *C. quilicii*).

In no‐choice tests, proportions of parasitized eggs were 0.15 ± 0.07 for *B. dorsalis* eggs, 0.19 ± 0.09 for *B. zonata* eggs, and 0.04 ± 0.03 for *C. quilicii* eggs and were significantly higher for *B. zonata* eggs than for *C. quilicii* eggs (*z* value = 3.639, *p* < .001, Figure 3).

Similarly, in choice tests, we observed a higher proportion of parasitized eggs for *Bactrocera* eggs than *C. quilicii* eggs in both species combinations: *B. zonata/C. quilicii* (*z* value = 7.543, *p* < .001) and *B. dorsalis/C. quilicii* (*z* value = −5.865, *p* < .001). In the condition *B. dorsalis/B. zonata*, the proportion of parasitized eggs was significantly higher for *B. zonata* eggs than *B. dorsalis* (*z* value = 4.532, *p* < .001, Figure 3).

#### 3.2.2 Host plant species

We did not observe a significant difference in the proportion of parasitized eggs between the colony of *F. arisanus* reared on *B. dorsalis* eggs and the colony reared on *B. zonata* eggs ($\chi_{1}^{2}$ = .262, *p* = .459).

**FIGURE 4:**
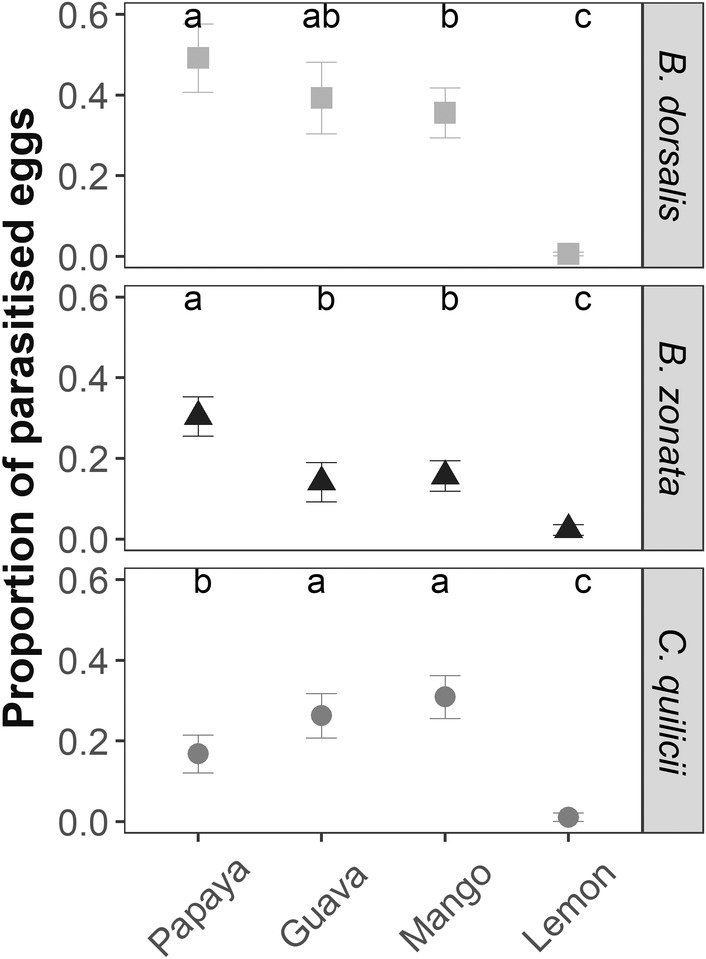
The proportion of parasitized eggs (mean ± SE) by *Fopius arisanus* according to host plant species on which eggs were deposited and fruit fly species. Different letters indicate a significant difference in parasitism rate among host plant species for each fruit fly species.

For all fruit fly species tested, eggs in lime fruit were the least parasitized. The proportion of parasitized eggs on lime fruit was 0.006 ± 0.004 for *B. dorsalis* eggs, 0.023 ± 0.013 for *B. zonata*, and 0.011 ± 0.010 for *C. quilicii* eggs. For *Bactrocera* species, eggs deposited in papaya were more parasitized than eggs deposited on mango and guava (only for *B. zonata*). On the contrary, for *C. quilicii*, eggs were more parasitized in guava and mango than in papaya (Figure 4).

## 4 DISCUSSION

With these multiple introductions of fruit fly pests and natural enemies, La Réunion is a good model to study how new interactions can impact ecological networks and tri‐trophic interactions. In particular, this is possible because of the long‐term field database of fruit samplings and fruit fly records (from 1991 to 2009 and 2018 to 2019) gathered in the UMR PVBMT, completed by bioassays performed in the laboratory. Our study shows an example of the impact produced when introducing a new species in a complex environment, with implications of tri‐trophic interactions between host plants, different fruit fly host species, and a parasitoid, and how the outcome on biological control of a species can be impacted. Our results are particularly interesting for the biological control of fruit flies in the context of the range expansion of *B. zonata* and *B. dorsalis*. In La Réunion, we point up that *F. arisanus* parasitism rate was highly variable according to the host plant species and location and almost doubled to 17.0% after *B. dorsalis* invasion. We demonstrated the capacities of *F. arisanus* experimentally to discriminate fruit substrate and eggs of different fruit fly species for oviposition. Surprisingly, *F. arisanus* preferred to lay eggs in *B. zonata* eggs than in *B. dorsalis* eggs. Finally, we discussed how field samplings and experimental results suggest a possible existence of indirect interaction.

### 4.1 *Fopius arisanus* parasitism rate

*Fopius arisanus* was released several times between the end of 2003 and 2005 to control *B. zonata* in La Réunion. Our results show that since these releases, the parasitism rate of *F. arisanus* has changed, as has its impact on fruit fly populations. To our knowledge, this is the first time that the parasitism rate of *F. arisanus* on *B. zonata* has been studied in the field. In 2005, individuals of *F. arisanus* were frequently found in fruit collected during regular sampling, but observed parasitism rates remained low (0.25%). Between 2006 and 2009, the parasitism rate fluctuated between 4.7% and 8.6%. *Fopius arisanus* was well established throughout the island. However, its impact on fruit fly populations appears to be negligible because we did not observe a significant difference between the main host plant's infestation rates and network indexes before and after the parasitoid introduction. Nevertheless, after the *B. dorsalis* invasion, we observed a significant increase in the parasitism rate of *F. arisanus* and a change in network structure. The global parasitism rate almost doubled to reach 17.0% (3012 individuals from 36 plant species) and its number of host plants (degree) increased. We also observed a decrease in cluster coefficient, nestedness, C. score and strength of *C. catoirii*, *C. quilicii* and *B. zonata*, suggesting a diminution of interactions between fruit flies (except *B. dorsalis*) and host plants. In La Reunion, a previous study shows evidence of a competitive displacement induced by *B. dorsalis* on other established species. A shift in host range and climatic niches was observed for *Bactrocera zonata*, *Ceratitis quilicii*, and *Ceratitis capitata* (Moquet et al., 2021). It's common that the invasion of a new species into a community modifies the network structure, often through the addition of a new node and new links (David et al., 2017). Our results suggested that *B. dorsalis* invasion modified both fruit‐fly/host plant, parasitoid/host plant, and probably parasitoid/fruit fly interactions.

However, the parasitism rate was highly variable according to the host plant species and location. In our results, this parasitoid was absent from 32 plant species infested by *B. dorsalis* or other generalist species, while the infestation rate reached 41 ± 17% for *Cananga odorata*. According to Moquet et al. (2021), in the plant species most infested by *B. dorsalis*, the parasitism rate by *F. arisanus* was 17 ± 3% for *M. indica*, 37 ± 2% for *T. catappa*, 16 ± 2% for *S. jambos*, 19 ± 1% for *P. cattleianum*, and 24 ± 2% for *P. guajava*. These values are low compared to parasitism rates observed in Hawaii and French Polynesia (Bess et al., 1961; Eitam & Vargas, 2007; van den Bosch & Haramoto, 1951; Vargas et al., 1993, 2007, 2012) where parasitism rates of *P. cattleianum*, *P. guajava*, and *T. catappa* were included between 41% and 73%. The global parasitism rate observed in our study (17%) is more similar to values recorded in Africa, where this parasitoid was introduced from Hawaii, and where the average parasitism rate varied according to studies from 1.7% in Mozambique to 14% in Senegal (Cugala et al., 2016; Gnanvossou et al., 2016; Ndiaye et al., 2015). The discrepancies in parasitism efficacy observed between the islands in the Pacific Ocean and Africa (including the Indian Ocean islands) could be linked to several factors. However, the host plants (very similar exotic species are found in these countries), and climatic conditions (the introduced areas cover a wide range of climatic conditions), do not appear to be the main explanatory factors for these differences. Other factors may be involved. First, when the *F. arisanus* population was initially introduced, only a few individuals were used. Consequently, the effective population size was small. This increased the effects of inbreeding and genetic drift, leading to a greater loss of genetic diversity and potentially affecting population fitness (Zaviezo et al., 2018). Another hypothesis could be that not all species of Tephritidae are suitable hosts for the parasitoid; and if eggs are laid in some non‐host species, it could be a dead‐end host for *F. arisanus* (Rousse et al., 2006). In Africa, in areas where it was recently introduced, a very different and broad community of Tephritidae species is found, which could also explain its reduced efficacy.

### 4.2 Host plant preference

We demonstrated the capacities of *F. arisanus* to discriminate fruit substrate for oviposition. For example, eggs deposited in lime (*C. aurantifolia*) were neglected in favor of other host plants. *Citrus* species have been widely recognized as poor hosts for fruit flies because of the chemical resistance in the peel (Greany et al., 1983; Papachristos & Papadopoulos, 2009; Ruiz et al., 2014). On the contrary, *F. arisanus* preferred guava and mango, hosts of high nutritional quality for polyphagous fruit fly species (Hafsi et al., 2016). Host selection by parasitoids seems to match the preference‐performance hypothesis. This hypothesis describes how the female selects the oviposition site to optimize the development of its progeny (Gripenberg et al., 2010). This trend was observed in parasitoids, including *F. arisanus* (Ayelo et al., 2017; Bautista & Harris, 1996), but it is less common in generalist species (Gripenberg et al., 2010; Monticelli et al., 2019). Moreover, the preference for a host plant varied according to the species of eggs deposited. In the no‐choice (tephritid host) experiment, *F. arisanus* preferred to lay eggs in the guava and mango when it was infested by *C. quilicii* eggs, the papaya and mango when it was infested by *B. dorsalis* eggs, and the papaya when it was infested by *B. zonata* eggs. *Fopius arisanus* adapted its preferences for the oviposition site according to the fruit fly species present. The preference‐performance hypothesis was not always confirmed. For example, *F. arisanus* preferred to lay eggs in papaya when *B. zonata* infested the fruit, whereas Hafsi et al. (2016) have shown that survivorship of *B. zonata* was very low on papaya. *Fopius arisanus* is classified as a generalist parasitoid, reported to be able to develop on over 80 host plant species from diverse families and on at least 35 host fly species belonging to Tephritidae (Gnanvossou et al., 2016; Nanga Nanga et al., 2019; Rousse et al., 2005). It has been suggested that the strength of the preference–performance relationships depends on the specificity of the diet (Gripenberg et al., 2010). In generalist species, insect behavior can be constrained by their ability to recognize specific cues of a fruit fly, host plant species, and a combination of the two.

Preferences of *F. arisanus* in the laboratory were consistent with field observations. We observed a higher parasitism rate on *C. papaya* and *P. guajava* (24 ± 2% for both), than on *M. indica* (17 ± 3%), and the parasitism rate was zero for *Citrus* species (except *Citrus tangerina*). While most studies focused on some highly parasitized species (mango, guava, tropical almond), we collected cultivated, ornamental, and wild host plant species. Some of these host plants had a significant infestation rate but a lower or null parasitism rate. For example, we found a parasitism rate of 2% for *Diospyros kaki*, *Ziziphus mauritiana* and 0% for *Musa* sp., *Prunus* sp., and *Pyrus* sp. It is essential to consider these species because they may represent a refuge for fruit flies. The ‘refuge theory’ proposed by Hawkins et al. (1993) predicts that if hosts occupy a large niche, parasitoids may fail to sufficiently reduce the host population's density for effective biological control. We were able to highlight refuge plants for *B. dorsalis*, *C. capitata*, and *C. quilicii*, but not *B. zonata* and *C. catoirii* (see the network shown in Figure 2). The absence of parasitism in some host plant species could result from the combination of sampling effort and the spatio‐temporal variations of the parasitism rate. Parasitoid populations can fluctuate as a function of climatic factors, host plant availability, and fruit fly density. Parasitoids can be attracted to highly infested patches or avoid already parasitized hosts (Aguiar‐Menezes & Menezes, 2001; Kitthawee, 2000). Models have shown that the spatio‐temporal heterogeneity in parasitism rate and the presence of host refuges can stabilize parasitoid‐host interactions (Briggs & Hoopes, 2004; Holt & Hassell, 1993). Nevertheless, empirical studies are required to understand the different parameters influencing parasitism rates in fruit fly parasitoids.

### 4.3 Parasitoid‐Tephritidae interaction

This study also shows how females of *F. arisanus* can discriminate between eggs of different fruit fly species. We have demonstrated that the preference for the host plant species varies depending on the fruit fly species infesting the fruit. Our original findings reveal that when the parasitoid had the choice between *B. dorsalis* and *B. zonata* eggs, it had a preference for the latter.

*Fopius arisanus* discriminate between the eggs of different fruit fly species for oviposition. Some tephritid species are known to deposit host‐marking pheromones near their oviposition sites (Scolari et al., 2021; Silva et al., 2012), which can act as kairomones for parasitoids (Prokopy & Webster, 1991; Roitberg & Lalonde, 1991). However, our study disregarded these marking pheromones because we moved eggs from the artificial support to the piece of fruit. Thus, only compounds present on the eggs can influence the observed behavior. Rousse et al. (2007) demonstrated that females of *F. arisanus* respond to kairomones emanating from the egg masses of Tephritidae, which could explain this behavior.

In choice and no‐choice experiments, *F. arisanus* preferred eggs of *Bactrocera* species to eggs of *C. quilicii*. This result was consistent with previous studies (Ayelo et al., 2017; Bautista & Harris, 1996; Mohamed et al., 2010; Rousse et al., 2006). It shows that *F. arisanus* can discriminate between fruit fly species. In this situation, the parasitoid preference is in line with performance. *F. arisanus* has a much higher survival rate when it parasitizes *B. zonata* (75.7%), than when it parasitizes *C. quilicii* (22.0%, Rousse et al., 2006). This could result from the long co‐evolution of these species. In its region of origin (Indomalayan region), as well as in regions of introduction (Hawaii), *F. arisanus* is found to parasitize *Bactrocera* species (Ramadan et al., 1992).

When *F. arisanus* had the choice between *B. zonata* and *B. dorsalis*, the parasitoid preferred *B. zonata* eggs. The natal host did not influence this preference because we observed the same result in both *F. arisanus* reared on *B. zonata* and on *B. dorsalis*. It is known that *F. arisanus* develop well in both these fruit fly species (Ayelo et al., 2017; Bautista & Harris, 1996; Mohamed et al., 2010; Rousse et al., 2006). *Fopius arisanus*, once introduced in 2003, was reared on *B. zonata*. After 14 years of successive generations on this host, it may have developed a preference for this host or its populations may have become better adapted to this host.

### 4.4 Indirect interactions

In our results, many parameters suggest that indirect interactions could exist between *B. zonata* and *B. dorsalis* via *F. arisanus*. First, both species were suitable hosts for *F. arisanus* (Harris & Bautista, 2001; Rousse et al., 2006) and share the same ecological niche in La Réunion (Moquet et al., 2021). Moreover, we observed a greater abundance of *F. arisanus* and a decrease in *B. zonata* infestation rate and the adult population just after the *B. dorsalis* invasion. This could be due to apparent competition, a mechanism that is mediated by density, whereby the greater abundance of one host allows an increase in parasitoid abundance and then has a negative impact on a second host species. In addition, although not tested here, trait‐mediated indirect interactions could add up to density‐mediated interactions if *B. dorsalis* induces changes in *F. arisanus* traits (morphological or behavioral) that could alter its interactions with *B. zonata*. Other studies show that field observation suggested an indirect effect even during the biological invasion (Chaneton & Bonsall, 2000). For example, (Settle & Wilson, 1990) documented the importance of indirect parasitoid‐mediated effects on the population decline of the grape leafhopper (Cicadellidae), *Erythroneura elegantula* Osborn, 1928, during an invasion of the variegated leafhopper, *E. variabilis* (Beamer, 1929), when an increase in the parasitoid *Anagrus epos* Girault, 1911 (Mymaridae) population was observed (Settle & Wilson, 1990).

Furthermore, the preference of *F. arisanus* for *B. zonata* could influence indirect interactions between the two *Bactrocera* species, with a shift towards *B. zonata*. If the natural enemy has a feeding preference for one type of prey, the interactions between the host species could be asymmetric, i.e. one prey species can have a negative effect on another prey species, while the reciprocal effect is near zero (i.e. amensalism). This situation is common (Brassil & Abrams, 2004; Chaneton & Bonsall, 2000) and could contribute to the significant decrease of the *B. zonata* population observed in La Réunion, following the *B. dorsalis* invasion (Moquet et al., 2021).

In La Réunion, *B. zonata* populations almost disappeared only 2 years after *B. dorsalis* was first detected. In 2022, no *B. zonata* was caught in traps installed around the island (Appendix S2). This observation could result from both direct and indirect competition between the two fruit fly species. Despite all the cases of invasion in fruit fly species, competitive exclusion is very rare. In fruit flies, the only case of exclusion was reported for *C. catoirii* in Mauritius because of pressure from successive invasions of different species over the years (Duyck et al., 2004, 2022). Although populations may be sufficiently abundant during biological invasions to cause interspecific competition (Duyck et al., 2022), many authors suggest that direct competition is not the determinant mechanism for phytophagous communities (Kaplan & Denno, 2007), which includes fruit flies (Clarke, 2016). On the contrary, more and more articles show that indirect interactions are common, such as apparent competition, which structures insect communities and produces similar patterns to those found when there is competition for resources (Bird et al., 2019; Frost et al., 2016; Morris et al., 2005; van Veen et al., 2006).

To conclude, with field sampling and experimental bioassays, our study suggests that direct and indirect interactions could significantly modulate the population of species in a tripartite network, even leading to the disappearance of a resident species. However, other experimental studies are necessary to confirm the part of indirect interactions in the network (Chaneton & Bonsall, 2000). In the context of invasion and biological control, understanding the outcomes of these multilevel interactions is necessary to predict the outcome of population control strategies.

## AUTHOR CONTRIBUTIONS

**Laura Moquet:** Conceptualization (equal); data curation (lead); investigation (equal); methodology (equal); validation (equal); writing – original draft (lead); writing – review and editing (equal). **Benoît Jobart:** Investigation (equal); methodology (equal); writing – review and editing (equal). **Romuald Fontaine:** Data curation (equal); investigation (equal); writing – review and editing (equal). **Hélène Delatte:** Conceptualization (equal); funding acquisition (lead); methodology (equal); supervision (lead); validation (equal); writing – review and editing (equal).

## Supporting information


Supinfo01

Supinfo02

## Data Availability

Data are available on CIRAD Dataverse https://doi.org/10.18167/DVN1/NYZ2NR (https://dataverse.cirad.fr/).
