# Supplementary material for: Tri‐trophic interactions among Fopius arisanus, Tephritid species and host plants suggest apparent competition
Source: Ecol Evol. 2023 Jan 11;13(1):e9742. doi: 10.1002/ece3.9742 (PMC9834009; doi:10.1002/ece3.9742)
Supplement: Supplementary file 1 — Supinfo01 [file ECE3-13-e9742-s001.docx]

**Tri-trophic interactions among *Fopius arisanus*, Tephritid species and host plants suggest apparent competition**

Moquet Laura^1*^, Benoit Jobart^1^, Romuald Fontaine^2^, Delatte Hélène^3^

**Appendix S1**: Other parasitoids found in La Réunion during samplings in 2018 and 2019

| **Parasitoid species** | **Number of individuals** | **Number of fruits with parasitoids** | **Host plant** | **Fruit fly species co-emerging with parasitoids** |
| --- | --- | --- | --- | --- |
| *Ealata* sp. | 25 | 9 | *Syzygium jambos* | *C. quilicii* |
|  |  |  | *Prunus persica* |  |
|  |  |  | *Prunus sp.* |  |
|  |  |  | *Syzygium samarangense* |  |
|  |  |  | *Psidium catlleianum* |  |
|  |  |  | *Ziziphus mauritaina* |  |
| *Psyttalia fletcheri* | 73 | 33 | *Coccinia grandis* | *B. dorsalis* |
|  |  |  | *Cucumis sativus* | *D. ciliatus* |
|  |  |  | *Cucurbita moschata* | *Z. cucurbitae* |
|  |  |  | *Cucurbita pepo* | *Z. demmerezi* |
|  |  |  | *Lagenaria siceraria* |  |
|  |  |  | *Lagenaria sphaerica* |  |
|  |  |  | *Momordica charantia* |  |
|  |  |  | *Sechium edule* |  |
| *Psyttalia insignipennis* | 2 | 2 | *Solanum americanum* | *N. cyanescens* |
|  |  |  | *Passiflora suberosa* | *C. capitata* |
| *Tetrastichus giffardianus* | 596 | 28 | *Eriobotrya japonica* | *B. dorsalis* |
|  |  |  | *Cucumis sativus* | *D. ciliatus* |
|  |  |  | *Cucurbita moschata* | *N. cyanescens* |
|  |  |  | *Mangifera indica* | *Z. cucurbitae* |
|  |  |  | *Momordica charantia* | *Z. demmerezi* |
|  |  |  | *Solanum melongena* |  |
|  |  |  | *Syzygium malaccense* |  |
|  |  |  | *Terminalia catappa* |  |
|  |  |  | *Ziziphus mauritaina* |  |
