# Supplementary material for: Tri‐trophic interactions among Fopius arisanus, Tephritid species and host plants suggest apparent competition
Source: Ecol Evol. 2023 Jan 11;13(1):e9742. doi: 10.1002/ece3.9742 (PMC9834009; doi:10.1002/ece3.9742)
Supplement: Supplementary file 2 — Supinfo02 [file ECE3-13-e9742-s002.docx]

**Tri-trophic interactions among *Fopius arisanus*, Tephritid species and host plants suggest apparent competition**

**Authors:** Laura Moquet^1*^, Benoit Jobart^1^, Romuald Fontaine^2^, Hélène Delatte^3^

**Appendix S2: Analyses of data from fruit fly epidemiological surveillance between 2015-2017 and 2022.** Data were collected in the context of the Biological monitoring of the territory - Surveillance of regulated or emerging organisms (SBT/SORE) piloted by the French Direction of Food, Agriculture and Forest (DAAF).

**Material and methods:**

Between 2015 to 2017, we used Maxi Trap (from SEDQ) with Methyl Eugenol as a male attractant and Deltamethrine under the lid as an insecticide. Lids with insecticide were changed every 3 months. 20 Traps were installed all around the island (Figure S1). They were monitored once a week.

After April 2017, 201 traps were installed around the island with a higher density near *B*. *dorsalis* infestation center (Figure 1). We used a Ceratipack trap adapted for *B. dorsalis* (with methyl Eugenol) instead of a Maxi Trap. Each trap was monitored approximately every 8 days.

In 2022, 10 traps with methyl Eugenol were re-installed at the entry points of the island (airports and harbours). This network was set up to detect the potential arrival of other flies sensitive to methyl eugenol. The traps used are recycled bottles with 4 entries fitted with Eppendorf tubes and were monitored once a week.

A Kruskal-Wallis test and pairwise comparisons using Wilcoxon rank sum test were realised to compare *B. zonata* number per day and per trap according to the period: Before *B. dorsalis* detection (until April 2017), after *B. dorsalis* detection (from May to August 2017) and now (2022).

**Result**

*B. dorsalis* was detected in April 2017. In the first months after *B. dorsalis* detection, the number of flies/trap/day was 0.04 ± 0.00. In 2022, we caught approximately 21.26 ± 18.61 *B. dorsalis* per trap and per day.

Before *B. dorsalis* detection, the mean number of *B. zonata* per trap and per day was 19.87 ± 0.49. Just after *B. dorsalis* detection, the number of *B. zonata* was significantly lower (P <0.001) and was, in mean, 2.68 ± 0.23. In 2022, no any *B. zonata* was caught (Figure S2).

**Conclusion**

In addition to the infestation rate from collected fruit, the trap network for fruit fly epidemiological surveillance allows us to confirm adult population trends. The first detection of *B. dorsalis* was in April 2017. We observed a decrease in the number of *B. zonata* caught just after *B. dorsalis* detection. In 2022, not any *B. zonata* was caught.

**
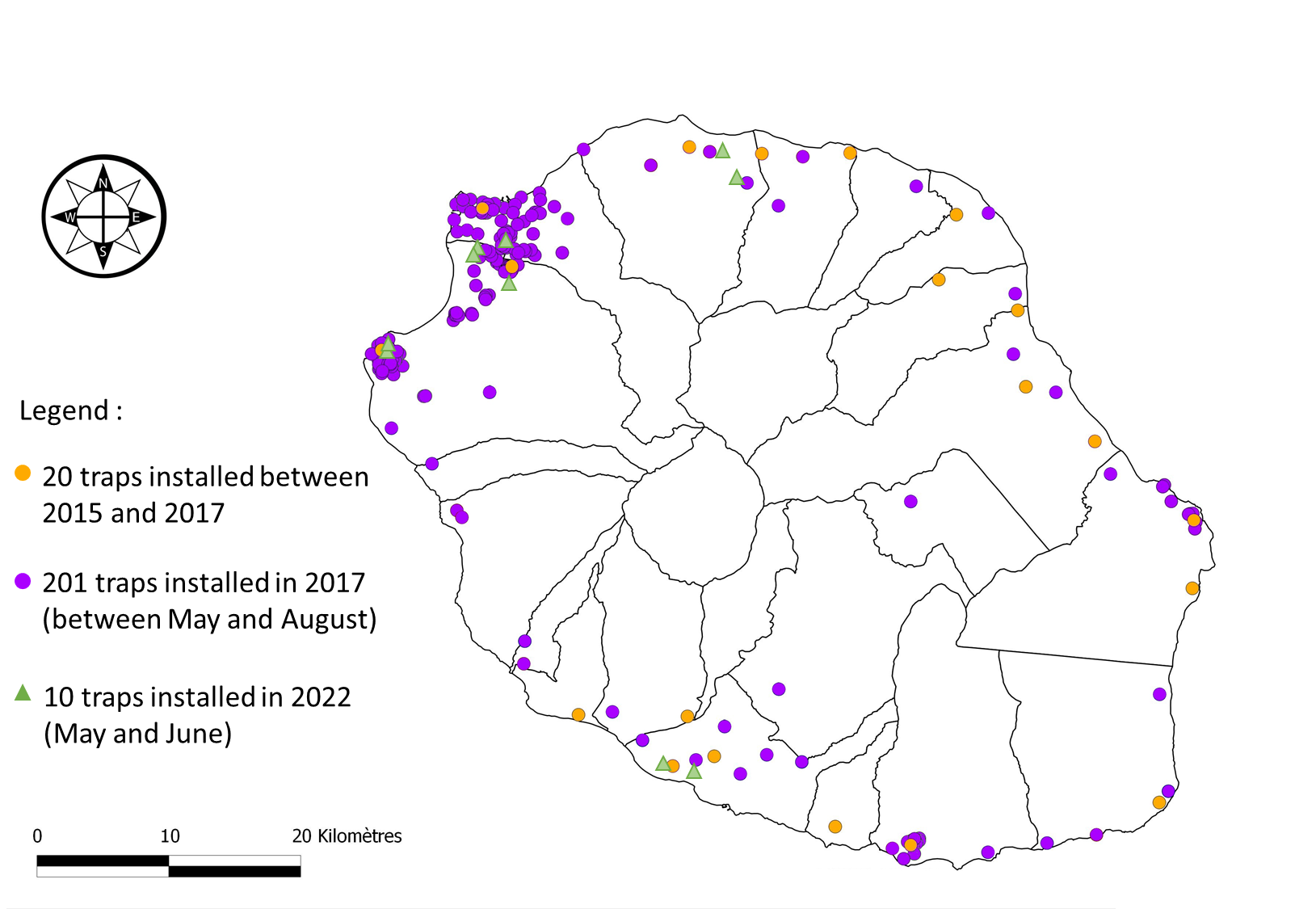
Figure S1**: Location of traps


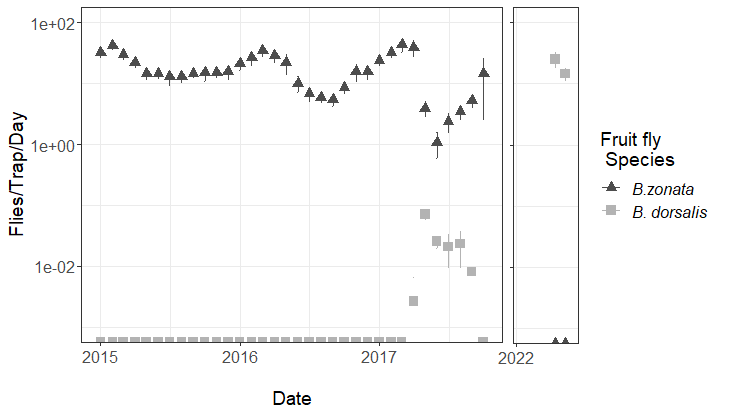


**Figure S2:** Number of fruit flies (B. dorsalis or B. zonata; average per month ± 1.96 se; Log scale) caught per trap and per day between January 2015 and August 2017, and in 2022 (May and June).
